# YouTube as a Source of Water Birth Information: An Analysis of Gaps, Misalignments, and Adherence With American College of Obstetricians and Gynecologists (ACOG) Guidelines

**DOI:** 10.7759/cureus.108351

**Published:** 2026-05-06

**Authors:** Antonia Oladipo, Natasha Malonza, Erika Fleming, Jamie Chen, Katherine M Collamore, Michel'le Bryant

**Affiliations:** 1 Obstetrics and Gynecology, Hackensack University Medical Center, Hackensack, USA; 2 Obstetrics and Gynecology, Hackensack Meridian School of Medicine, Nutley, USA

**Keywords:** health misinformation, maternal health care, maternal medicine, water birth, youtube videos

## Abstract

Background

Water birth is increasingly popular, yet debates persist regarding its safety and efficacy. There is a need to assess the accuracy and sentiment of publicly available water birth content, and it is essential that water birth content be accurate to support informed decision-making.

Objective

To identify whether certain YouTube video characteristics play a role in determining whether the contents of the video are aligned with the published recommendations set forth by the American College of Obstetricians and Gynecologists.

Materials and methods

An analysis of the top 100 English-language YouTube videos on “water birth” sorted by “most viewed” was conducted on March 9, 2023. After applying inclusion and exclusion criteria, a final set of videos met the inclusion criteria and were included in the analysis. Video characteristics were recorded. Video accuracy was assessed against 16 ACOG water birth guidelines. Scores: 1 (accurate), 0 (not mentioned), or -1 (inaccurate). Transcripts were analyzed using MonkeyLearn Sentiment Analyzer to determine sentiment.

Results

Of the videos analyzed, the majority were neutral in their accuracy, while a smaller proportion were deemed accurate or contained inaccuracies. Critical safety topics, such as umbilical cord avulsion or neonatal infection risks, were almost universally omitted. Videos created by healthcare professionals demonstrated greater accuracy, while personal vlogs were predominantly neutral. Sentiment analysis revealed that most videos conveyed a negative sentiment, followed by positive and then neutral sentiment. Notable geographic disparities were observed, with North American content exhibiting greater emotional polarization compared to international content.

Conclusion

Most widely viewed YouTube content on water birth lacks alignment with ACOG guidelines, particularly regarding risk communication, posing misinformation risks.

## Introduction

Water birth is defined as immersion throughout the complete birthing process, often combined with water immersion during labor. Water immersion can occur during labor or delivery, whereas water birth involves immersion throughout the entire birthing process [1,2]. Proponents of water birth suggest that submersion for labor and delivery reduces maternal pain and analgesia use, while critics highlight potential risks, including neonatal infection, aspiration, and umbilical cord avulsion [3-5]. While there is currently no definitive evidence to decisively reject or support the decision to give birth in water, the interest in water birth has been increasing [2,4]. Notably, some studies report a 30% increase in online searches for alternative birth options five years ago, a trend that has since remained consistently elevated [6].

This growing interest coincides with a broader trend: 92% of pregnant women use the internet to access prenatal health information on social media platforms like YouTube [7,8]. This suggests that the content analyzed here has potential reach and influence. Given the growing interest and the variety of perspectives and opinions on waterbirth efficacy and safety, it is crucial to evaluate the information about water birth available to the public, given the potential for misinformation to influence decision-making. Misinformation relating to pregnancy and birth can have negative consequences, such as influencing maternal decision-making, increasing anxiety, or encouraging unsafe practices. 

The American College of Obstetricians and Gynecologists (ACOG) published recommendations on immersion in water during labor and delivery, which state that water immersion during the first stage of labor may be safe, while delivery under water remains controversial [1]. Therefore, it is essential to evaluate the content of these popular videos to determine whether they align with established published recommendations set forth by the ACOG, and further evaluate if there are specific YouTube video characteristics present in the videos that are aligned. 

Previous research has explored online misinformation in women’s reproductive health and pregnancy, including inaccurate promotion of unproven interventions [9]. However, there has been little investigation into YouTube water birth content specifically, a gap this study aims to address.

Furthermore, sentiment analysis, a natural language processing technique, was used to assess the overall tone of the video, allowing evaluation of how the video’s content might be perceived emotionally by viewers. Sentiment analysis was included to explore the tone of discussion around water birth, as emotional framing of information may influence decision-making.

As the process of sharing labor and delivery experiences online gains popularity, it is important to understand whether discussions of water births on YouTube are in agreement with the currently published recommendations set forth by ACOG, and to identify the prevailing sentiments in popular videos that influence the public sphere. While professional sources might be expected to adhere to guidelines, personal videos may prioritize sharing experiences rather than comprehensive risk communication. Nonetheless, given the widespread reach of these videos, it remains important to analyze both personal and professional content.

## Materials and methods

**Figure 1:**
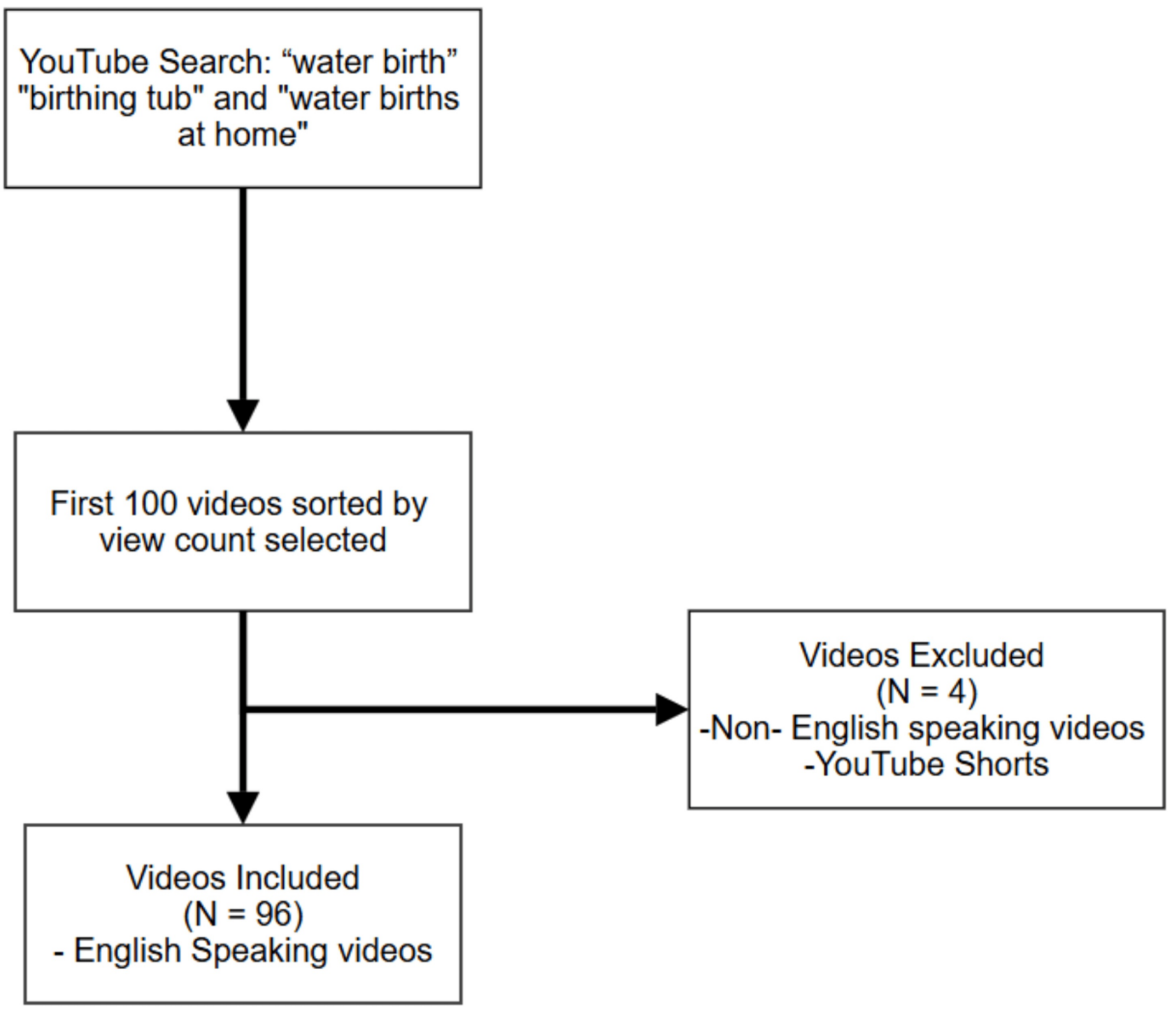
YouTube Video Selection Process Of those 100 videos, 96 videos met the inclusion criteria and were examined in this study. 4 videos that were either YouTube ‘shorts’ or non- english speaking were excluded.

This retrospective study featured a YouTube search using multiple keywords: 'water birth,' 'birthing tub,' and 'water births at home' highlighted by the use of a new profile, private internet browser, and "most viewed" filter. The first 100 most viewed videos were selected to ensure a diverse sample of popular content. Of those 100 videos, 96 videos met the inclusion criteria and were examined in this study (Figure 1).

Additional video metrics, including likes and views (Table 1), were obtained. This data was sorted by geographic distribution, video type, and content creator characteristics, including whether the creator was a health professional, midwife, doula, or individual user (Appendix 1). This approach helped to ensure a comprehensive analysis of water birth content available on YouTube, focusing on both the accuracy of the information provided and the tone conveyed. This study was exempt from institutional review board approval because it utilized publicly available data and did not include human subjects.

**Table 1:** Additional Video Metrics Collected

Additional Video Metrics Collected
Likes
Views
Amount of Comments
Length/Duration
Year Published
Subscribers (at time of data collection)
Age Restricted

Three reviewers scored videos in accordance with published guidelines from ACOG [10,11] and created an evidence-based “Water Birth Rubric” (Appendix 2). Alignment was assessed in line with the broader evidence review presented within the sixteen distinct recommendations by ACOG (Table 2). The rubric did not undergo formal validation processes and should be considered an exploratory instrument for categorizing content alignment with ACOG guidance. Each video was assigned a score of 1 if it described the information covered in the guideline and presented it in accordance with the ACOG recommendations, 0 if the information in the recommendations was not mentioned, and -1 if it was included but presented incorrectly. A total summative score was calculated with a maximum accuracy of 16, neutral accuracy of 0, and a maximum inaccuracy of -16.

**Table 2:** Categories Each Video was Assessed and Scored In

ACOG Recommendations	
Neonatal Diving Reflex	Methods of Delivery
Absence of Neonatal Benefits	Less Episiotomy
Neonatal Infection Risk	Shorter Duration of Labor
Neonatal Aspiration Risk	Less Analgesia Use
Umbilical Cord Avulsion Risk	Increased Maternal Satisfaction
Lack of Maternal Health Benefits	Candidate Selection for Water Birth
Lack of Maternal Health Risks	Infection Prevention Protocols
Absence of Increased Neonatal Risk	Monitoring for Contingency Plans

To analyze the sentiments of each of the selected videos, MonkeyLearn Sentiment Analyzer (MonkeyLearn, Inc., San Francisco, USA), a specialized machine learning platform designed for granular sentiment analysis that produces results with a confidence interval, was used. Video transcripts, if available, were input into the online MonkeyLearn Sentiment Analyzer. Results were categorized as neutral, positive, or negative. The confidence level of the sentiment was recorded. Chi-square goodness-of-fit and independence tests were performed to assess observed proportions of sentiments compared to the expected distribution. In addition to sentiment analysis of video transcripts, video thumbnails were analyzed. A supportive thumbnail is defined as a video thumbnail that includes a figure visibly providing comfort or emotional support to the birthing parent, such as through touch, presence, or a reassuring gesture. This characteristic is evaluated by human graders to determine whether the thumbnail depicts a supportive individual engaged in a comforting interaction with the birthing person.

For accuracy scoring, a score of 0 or neutral indicates that the video did not mention the specific ACOG recommendation, neither affirming nor contradicting it. This scoring category reflects the absence of information, not a balanced presentation of evidence. For sentiment analysis, 'neutral' refers to emotional tone lacking strong positive or negative valence, as determined by the MonkeyLearn algorithm. 

To assess variations of accuracy and sentiment across different video characteristics, we conducted stratified analyses comparing content creator types (health professionals, midwives/doulas, and individual users), geographic distributions (North America, Europe, and other regions), and video formats (educational, personal vlogs, and promotional content). Chi-square goodness-of-fit tests were employed to examine deviations from expected sentiment distributions. Chi-square tests of independence to evaluate relationships between sentiment outcomes and three key variables: creator credentials, geographic origin, and adherence to ACOG clinical guidelines. All statistical tests were two-tailed with significance set at p<0.05. This multi-layered approach enabled the detection of systematic biases in how different stakeholder groups portray water birth experiences and recommendations.

## Results

Of the first 100 most viewed English-speaking videos, 96 videos met the inclusion criteria. Video characteristics such as video performance metrics, video type, content creator, and channel association characteristics were then analyzed by geographic region (Table 3). Key findings revealed that the majority of videos were from North America (n=76, 79.2%), and these videos had the highest average views. The videos from Asia had the highest average likes despite having only three videos. Videos from both Europe and Australia showed moderate engagement with approximately 3 million average views, with videos from Europe having fewer average likes than videos from Australia.

**Table 3:** Video Characteristics by Geographic Region

Country	# of videos	Average Likes	Average Views
Australia	6	26,133	3,469,175
Africa	2	5,900	1,087,595
Asia	3	89,133	4,015,058
Europe	8	30,538	2,923,356
North America	76	45,836	5,915,588
South America	1	5,900	5,286,729

**Figure 2:**
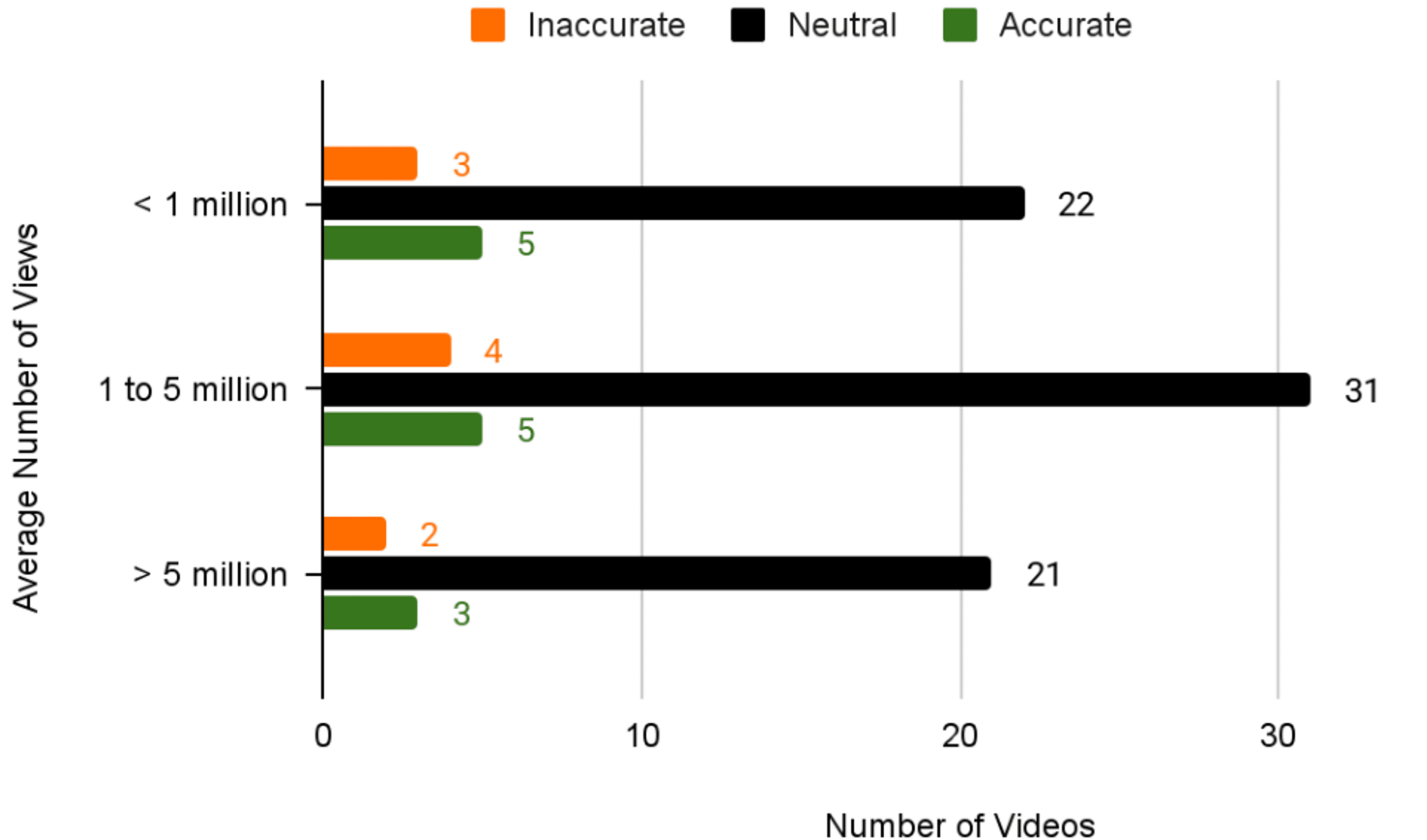
Accuracy by Average Number of Views

Across all viewership levels, neutral-accuracy videos consistently represented the majority of content (Figure 2). For videos with under 1 million views, 73% (n=22) were neutral-accuracy, while 10% (n=3) videos contained inaccurate information and 17% (n=5) were deemed accurate. Videos with 1 to 5 million views showed similar proportions, with 78% (n=31) neutral-accuracy videos (n=31), followed by 10% inaccurate videos (n=4) and 12% accurate ones (n=5). The most popular videos with more than 5 million views had the highest proportion of neutral-accuracy content, 81% (n =21), and the lowest accuracy rate, 11.5% (n=3), with 8% (n=2) of videos containing inaccuracies. 

**Figure 3:**
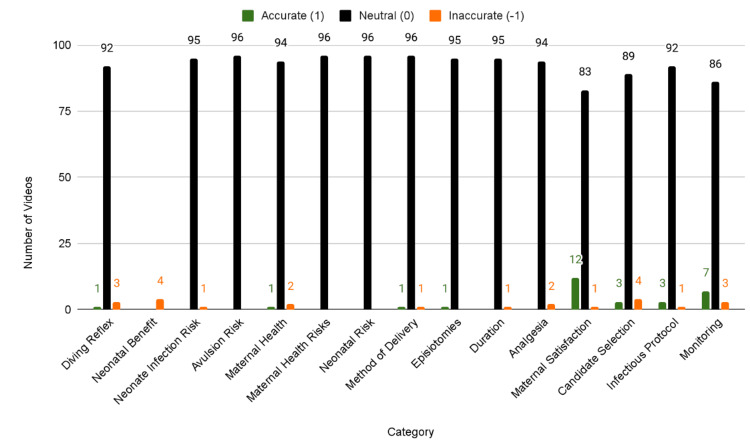
Spread of Water Birth Rubric Score Adherence

In terms of videos that adhere to the ACOG-derived “Water Birth Rubric,” 75% (n=72) scored neutral, 15.6% (n=15) accurate, and 8.3% (n=8) inaccurate (Figure 3, Table 4). 

**Table 4:** Spread of Water Birth Rubric Score Adherence

Category	Inaccurate (-1)	Neutral (0)	Accurate (1)
Diving Reflex	3	92	1
Neonatal Benefit of Waterbirth	4	92	0
Neonate Infection Risk	1	95	0
Avulsion Risk	0	96	0
Maternal Health Benefits	2	94	0
Maternal Health Risks	0	96	0
Neonatal Risk	0	96	0
Method of Delivery	0	96	0
Epesiotomies	0	95	1
Duration	1	95	0
Analgesia	2	94	0
Maternal Satisfaction	1	83	12
Candidate Selection	4	89	3
Infectious Protocol	1	92	3
Monitoring	3	86	7

**Figure 4:**
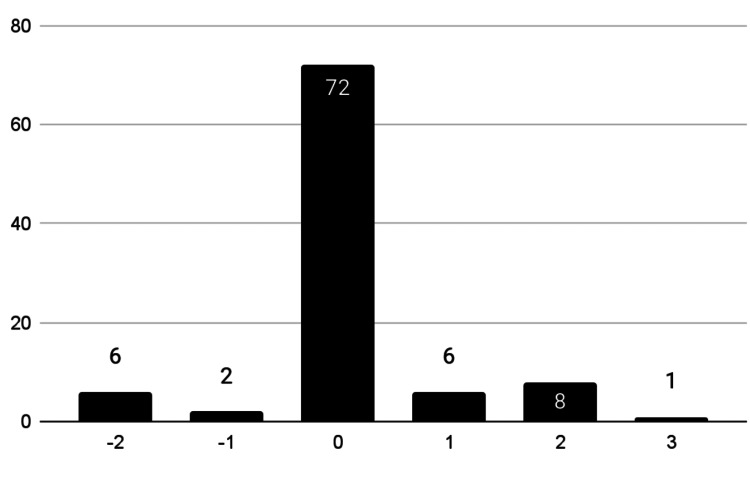
Summative Adherence Score Distribution

No videos addressed alternative methods of delivery, umbilical cord avulsion risk, maternal health, or neonatal infection risk. ACOG score distribution did not differ significantly by region. The summative scoring analysis revealed a majority (75%, n=72) achieved a neutral-accuracy score of 0, demonstrating neither accurate nor overtly inaccurate information. 16.7% (n=16) scored in the positive range (1-3), indicating some degree of alignment with ACOG recommendations. 8.3% (n=8) that received negative scores (-2 to -1), representing content that actively contradicted or misrepresented the ACOG recommendations (Figure 4). 

**Figure 5:**
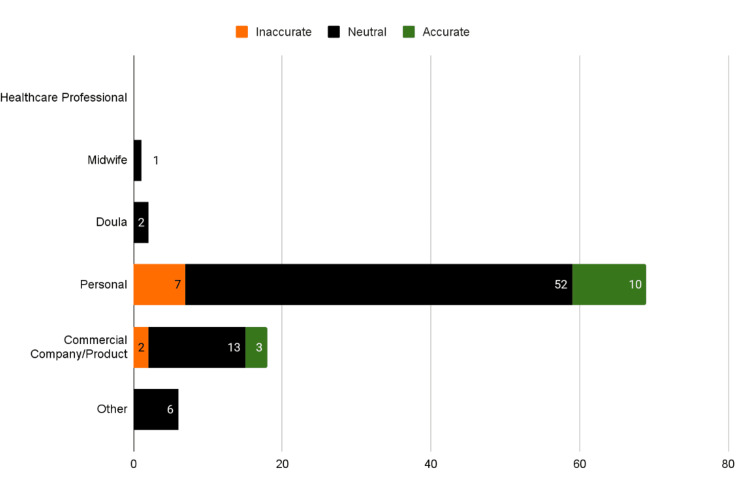
Accuracy by Content Creator

Commercially produced content demonstrated the highest adherence to ACOG waterbirth guidelines, while personal accounts were predominantly neutral-accuracy or inaccurate (Figure 5). When examining content creators, healthcare professionals (midwives and doulas) showed minimal inaccuracies, whereas personal accounts comprised the majority of neutral-accuracy (54.2%, n=52) or inaccurate (7.29%, n=7) videos. Commercial productions, despite consisting mainly of neutral-accuracy (13.5%, n=13), maintained overall accuracy compared to other creator types.

**Figure 6:**
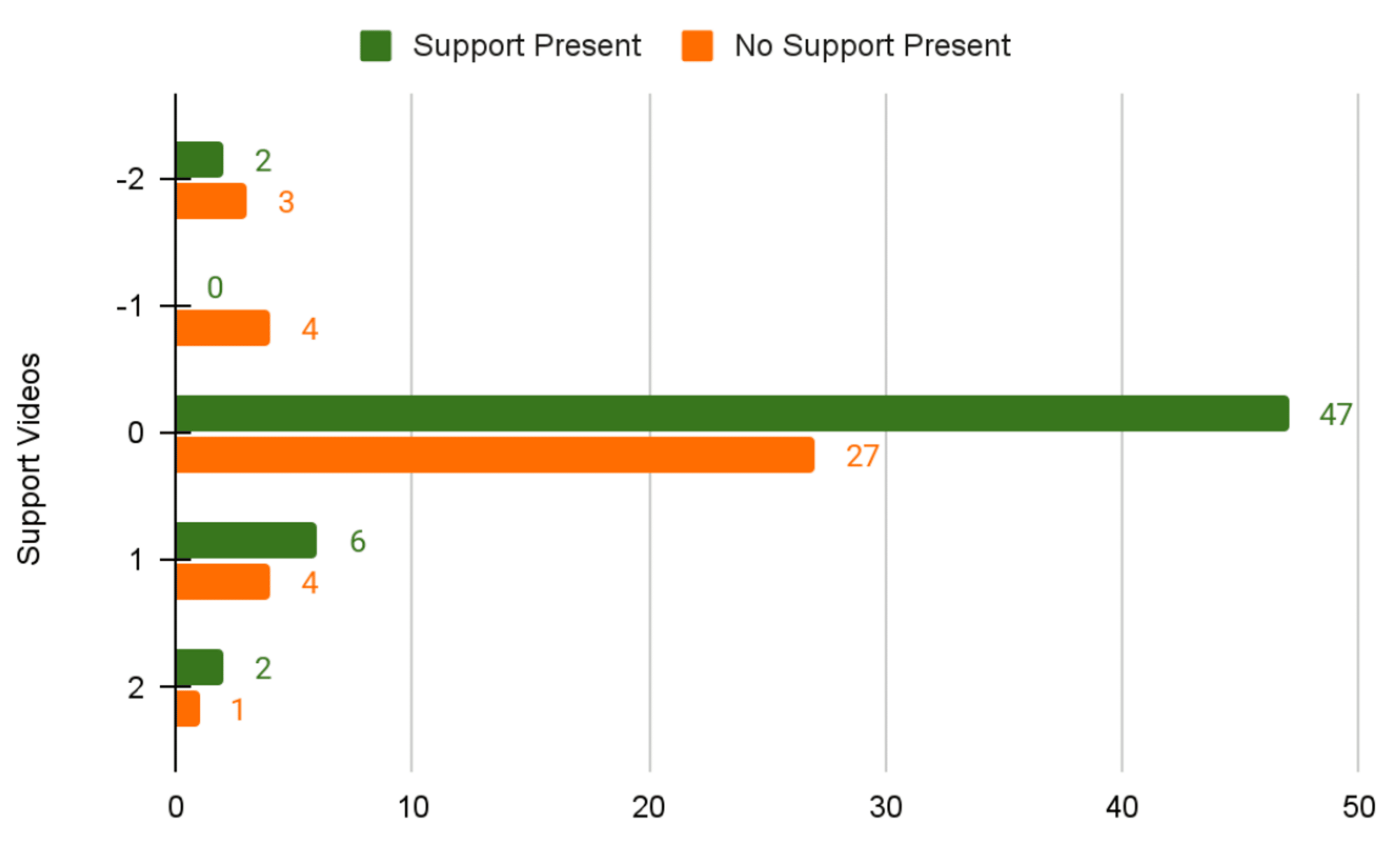
Comparing Supportive Figures and Content Accuracy

Supportive thumbnail imagery emerged as a reliability marker where the presence of supportive thumbnail imagery correlated with lower inaccuracy rates and higher neutral-accuracy content proportions (Figure 6). Videos featuring supportive figures in their thumbnails were significantly less likely to contain inaccurate information (X² (df=1, N=96)=13.2, p=0.0003).

**Figure 7:**
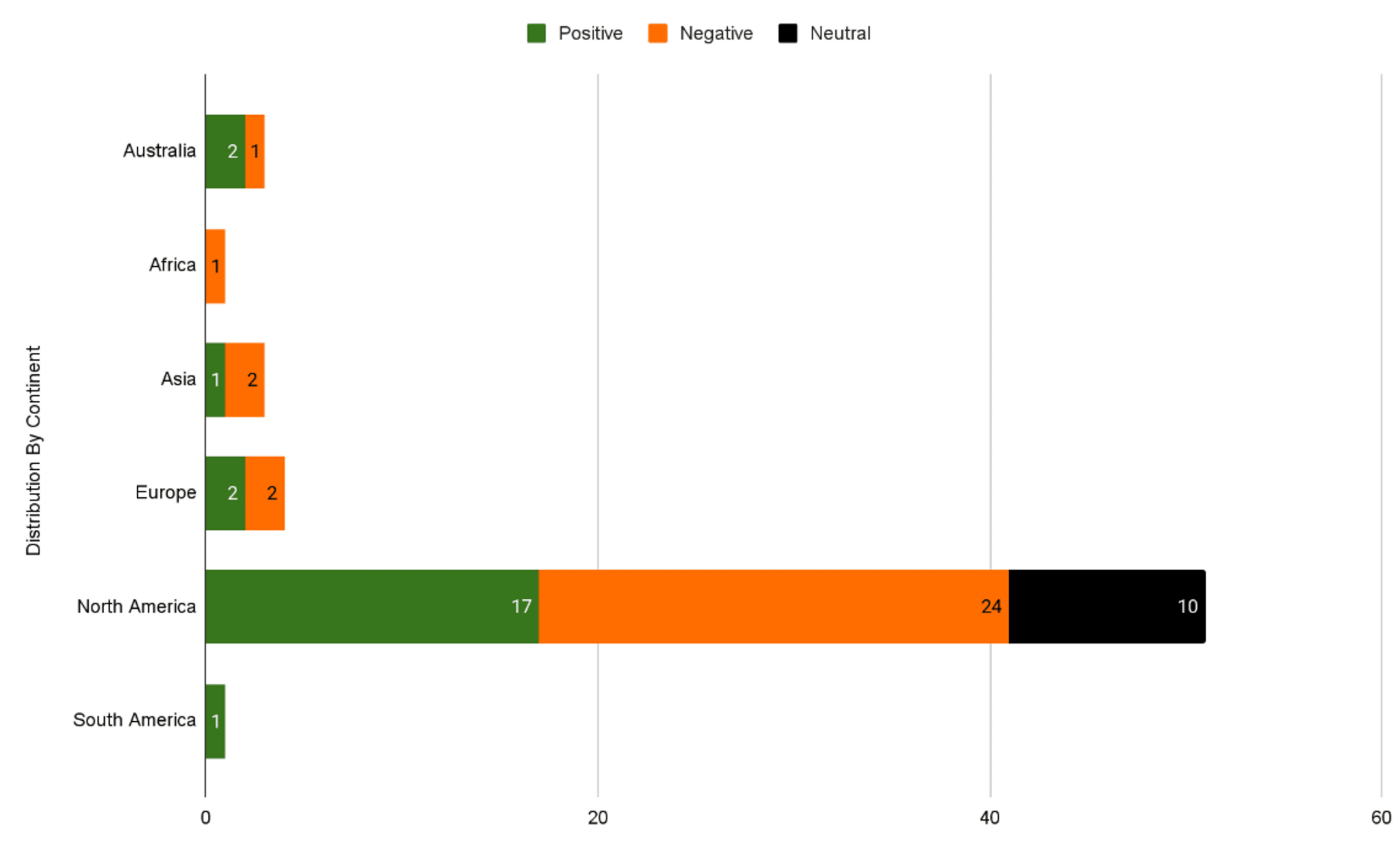
Breakdown Sentiment Score of Videos by Continent

The distribution of video sentiments differed significantly between North American and non-North American content (Figure 7). Positive sentiment was observed in 30.9% (n=17) of North American videos compared to 27% (n=6) of international videos, a statistically significant disparity (X² (df=1, N=75)=5.24, p=0.022). Conversely, negative sentiment appeared nearly twice as frequently in North American videos (47%, n=24) versus international videos (27%, n=6), with this difference being highly significant (X² (df=1, N=64)=10.86, p=0.001). All reported comparisons met the threshold for statistical significance (p<.05). Neutral sentiment showed an inverse pattern, comprising 19.6% (n=10) of North American videos but 45% (n=10) of international content.

## Discussion

This study evaluated 96 YouTube water birth videos against ACOG guidelines, finding that only 15.6% (n=15) were fully accurate, while 77.1% (n=74) were neutral and 8.3% (n=8) contained inaccuracies. Critical risks like umbilical cord avulsion and neonatal infection were absent in 96% (n=92) of videos. Healthcare professional-created content was more accurate than personal vlogs, which dominated neutral-accuracy or inaccurate content. Sentiment analysis revealed 47.6% (n=46) of videos were negative, 36.5% (n=35) positive, and 15.9% (n=15) neutral-sentiment, with North American content showing greater polarization (47%, n=24 negative vs. 27%,n=6 internationally, X² (df=1, N=64)=10.86, p=0.001). Supportive thumbnail imagery correlated with fewer inaccuracies (X² (df=1, N=96)=13.2, p=0.0003).

Primarily, this study sought to assess the accuracy of YouTube videos related to water birth, primarily assessing alignment with clinical guidelines established by ACOG, an essential investigation given YouTube’s growing influence on perinatal decision-making. While interest in water birth is prevalent, as depicted by the average number of views and comments on videos, a substantial proportion of online video content lacks clinical accuracy. 13.5% (n=13) of videos presented accurate information limited to maternal satisfaction and monitoring protocols. The majority of videos, 77.1% (n=74), were neutral in terms of guideline adherence. 

This “neutrality paradox”, where omissions masquerade as balanced content, represents a concerning absence of critical safety information rather than balanced coverage, as 96% (n=92) of videos failed to address risks like umbilical cord avulsion, neonatal infection, or maternal health complications. Data showed that the videos with one to five million views, which had significant reach and influence, fell into this “overall accurate but partially inaccurate” category. 10% (n=4) of these videos contained inaccurate information, highlighting the need for a more comprehensive evaluation of the content patients are consuming.

The correlation between creator type and content accuracy demonstrates a clear trend: videos by healthcare professionals and commercially produced content had fewer inaccuracies than personal vlogs, highlighting a potential risk for misinformation among general viewers seeking firsthand experiences. Work by Karatas et al., on the quality of YouTube videos on back pain during pregnancy, revealed that videos produced by healthcare professionals were of higher quality than those by non-professionals, situating our findings within a broader pattern of YouTube pregnancy content [10]. Videos created by healthcare professionals had 3.2 times higher odds of being accurate when compared to those created by laypersons [8]. This aligns with our findings, suggesting that health-related information on YouTube is often incomplete or inaccurate, especially when disseminated by non-professionals 

Although a statistically significant association between supportive imagery in thumbnails and decreased misinformation was noted (X² (df=1, N=96)=13.2, p=0.0003). This suggests an interesting trend that thumbnails and visual framing may impact perceived reliability of the content, potentially reflecting greater production input or institutional oversight. This raises a hypothesis that emotional framing may coexist with reduced attention to clinical accuracy, but our study design did not establish whether sentiment causes misinformation, whether misinformation generates emotional responses, or whether both reflect underlying creator characteristics

The relationship between content accuracy and sentiment reveals critical insights about viewer engagement with water birth information. The positive sentiments expressed in videos may relate to the reported benefits of water immersion during labor, such as reduced pain and anxiety. However, the consistent lack of discussion around risks, such as umbilical cord avulsion or neonatal infection, may skew perception, failing to present a balanced view of both benefits and potential harms. This echoes research on misinformation in the reproductive space, showing that recommendations about the administration or use of medical interventions that go against or are not supported by current medical practice guidelines are some of the most frequently occurring types of misleading claims [9]. For example, positive sentiment present in many videos coexisted with neutral-accuracy or inaccurate clinical content. Ultimately, this highlights a concerning disconnect where emotional appeal may mask informational gaps. Interestingly, despite frequently encountering misleading information online, pregnant women generally rate digital resources as 'good' and 'useful' for meeting their information needs, suggesting emotional engagement may override accuracy considerations in perceived value [8]. 

The secondary aim of this study was to assess the sentiments present in water birth videos. 47.6% (n=46) of videos had a negative sentiment, 36.5% (n=35) of videos had positive sentiment, and 15.9% (n=15) had neutral sentiment. The positive sentiments expressed in many of the videos may relate to the reported benefits of water immersion during labor, such as reduced pain and anxiety. Further analysis revealed an interesting geographic distribution: North American videos demonstrated higher emotional polarization compared to international content. Comparable polarization has been observed in discourse on social platforms by Van Bavel et al., with research by John et al., revealing broader sociocultural dynamics, such as political and ideological campaigns, may shape how misinformation on reproductive health can spread [9,12]. 

Negative sentiment appeared in 47% (n=24) of North American-based videos versus just 27% (n=6) internationally (X² (df=1, N=64)=10.86, p=0.001), while positive sentiment showed a smaller but still significant difference (30.9% vs 27%, X² (df=1, N=75)=5.24, p=0.022). These findings suggest that the North American-based content may present more emotionally polarized narratives, potentially amplifying both enthusiasm and concern around water birth in ways that diverge from international trends. This finding may be partially influenced by the exclusion criteria of non-English speaking videos, as we may not have collected a representative sample of videos from each non-English speaking region. The sentiment differences observed may stem from several factors. Geographic comparisons should be interpreted with caution due to uneven sample sizes across regions. While North America contributed 76 videos enabling robust statistical comparisons, regions such as South America (n=1), Africa (n=2), and Asia (n=3) were represented by too few videos to support reliable inferences. The significant differences observed between North American and international content may obscure important variation among non-North American regions.

Sentiment and accuracy patterns varied notably with content popularity, revealing trends in how information is presented across different audience sizes. While neutral-accuracy videos dominated all viewership levels, making up 73% (n=22) of videos under 1 million views, 78% (n=31) of 1-5 million views, and 81% (n=21) of 5 million views, this apparent balance masked significant informational gaps. The most viewed videos (> 5 million views) demonstrated the highest proportion of neutral-accuracy content (81%, n=21) yet the lowest accuracy rate (11.5%, n=3), suggesting that neutrality often reflects omission rather than true balance. 

A pattern emerged when examining polarized sentiment in videos: positive or negative sentiments tended to have more partial inaccuracies, especially when discussing risks. This aligns with research showing that emotionally charged content, even if inaccurate, is more memorable and shareable [13]. Among mid-tier viewership (1-5 million views), where neutral-sentiment videos comprised 78% (n=31) of content, the remaining 22% (n=9) of polarized videos accounted for nearly all clinically significant omissions regarding neonatal risks. This suggests sentiment alone cannot predict reliability, necessitating more robust assessment tools. Systematic reviews found that medically accurate visuals improved retention of information compared to text-only formats [14]. However, emotionally charged imagery without clinical grounding, common in personal vlogs, can increase retention of inaccurate content, underscoring the need for expert-reviewed visual aids. In the context of water birth, this creates a concerning scenario where dramatic narratives may disproportionately shape viewer perceptions, while more balanced, evidence-based content receives less engagement. YouTube’s recommendation algorithms may be prioritizing emotionally provocative content based on engagement, as evidenced by the disproportionate reach of polarizing videos in the 5 million+ view category, where accuracy rates drop to 11.5%. Amplification of emotionally charged content could explain why polarizing videos achieve disproportionate reach while accuracy declines [13]. This could also be due to YouTube’s primary appeal as an entertainment source, making users less wary of inaccuracies [15]. 

Our findings expose flaws in current health communication paradigms by raising concerns about the public-health impact of online birth information. Misinformation, whether overt or through omission, could lead to uninformed decisions, particularly when personal vlogs, which make up 54.2% (n=52) of neutral/inaccurate content, dominate search results. These results converge with calls for clinician-led, evidence-based video resources to counterbalance experiential but incomplete narratives [15]. A narrative review evaluating reproductive health claims by John et al. found that 33% of claims misrepresented evidence-based interventions by linking them to unsubstantiated risks, while 23% provided recommendations that conflicted with clinical guidelines [9]. One example cited in the review showed that female community college students who believed that contraception causes infertility were significantly less likely to use hormonal contraception. Such findings highlight the broader implications of misinformation on reproductive decision-making. In the context of childbirth, misinformation about risks could lead to harmful decisions, and even partially inaccurate content may mislead viewers. These findings suggest that most water birth content on YouTube may not adequately inform the public in a clinically reliable way, despite its widespread consumption. 

The clinical implications of these sentiment patterns are substantial. While emotional storytelling can make health information more relatable, our findings suggest that in perinatal contexts, strong sentiment, particularly when unaccompanied by clinically accurate information, may inadvertently mislead viewers. This is particularly true for North American audiences, who appear to be exposed to more polarized content than their international counterparts. From a public health perspective, the findings highlight the importance of improving digital health literacy and integrating accurate, accessible information into popular platforms like YouTube. Given the rising popularity of water births and the growing reliance on social media for health education, clinicians and organizations like ACOG may benefit from developing targeted video content to address gaps in public knowledge.

While this study provides novel insights into sentiment patterns of water birth content, several limitations should be acknowledged. The evidence-based rubric was developed using ACOG Committee guidelines, as previously stated; however, these guidelines were published nine years ago, and some of the cited evidence is outdated or limited. ACOG itself acknowledged that the available evidence was insufficient to draw firm conclusions on the risks and benefits of second-stage water birth.

While we used multiple search terms ('water birth,' 'birthing tub,' 'water births at home'), we acknowledge that other variations such as 'birth in water,' 'natural water birth,' or 'labor in water' may have yielded additional relevant videos. The selected terms were chosen based on preliminary searches identifying them as the most common user search patterns, but this approach may have excluded some relevant content. Future studies should employ more exhaustive search strings or use YouTube's auto-complete suggestions to identify the full range of user search terms.

Our analysis uncovered a fundamental challenge in evaluating health communication quality: the operationalization of "accuracy" in health communications, specifically perinatal health communications. While some videos were categorized as “accurate” based on their overall positive score in adherence to ACOG guidelines, several of these still contained inaccurate or omitted recommendations, revealing that this binary scoring system failed to capture critical nuances. For instance, a video could score +2 by correctly addressing maternal satisfaction and method of delivery while scoring -1 by incorrectly presenting neonatal infection risks, overall earning a +1, a combination that meets technical criteria for accuracy but remains clinically inadequate. Can a video that misrepresents nearly half of the clinically endorsed information still be considered truly accurate? 

Additionally, in our analysis of content sentiment, we acknowledge that some negative remarks may not directly criticize water birth but rather reference individual circumstances. Nonetheless, such negative sentiments may contribute to an emotionally charged tone that enhances the content’s popularity, regardless of the content’s accuracy. The use of YouTube’s “most viewed” filter may over-represent popular opinions while under-representing newer or niche content. Moreover, the rubric developed for ACOG alignment, while structured, may not account for implicit or inferred messaging within the videos. The rubric is only looking at the presence or absence of the limited evidence provided in the ACOG guideline, and doesn't take into account the more nuanced variations that may exist. Sentiment analysis using MonkeyLearn without inter-rater reliability checks, which measure the degree of agreement between 2+ independent raters, may introduce measurement bias. Though methodologically sound, MonkeyLearn is still reliant on natural language processing tools that may not fully capture nuances in tone or context. 

Inter-rater reliability was not performed due to time and resource constraints, which represents a methodological limitation. Video evaluation was completed collaboratively rather than independently. While consensus discussions helped standardize interpretations, this approach does not provide readers with confidence that the scoring would be reproducible by other researchers. As the video content and platform context have evolved substantially, recalculation of reliability metrics is not feasible at this stage. Future studies should incorporate formal reliability testing with independent ratings before consensus resolution. Without validation through independent human coding of a sample subset, we cannot fully quantify potential misclassification rates for sentiment labels. Future studies would benefit from a hybrid approach: using MonkeyLearn for initial screening followed by human verification of ambiguous cases.

While YouTube remains a powerful tool for health education, the current state of water birth content demonstrates a need for clearer, more accurate information and stronger collaboration between medical professionals and content creators. Addressing these gaps is essential to ensure that public health messaging keeps pace with digital consumption trends. Future evaluations may benefit from more granular scoring systems or weighted criteria that reflect the clinical significance of specific information, rather than equalizing all elements of the guidelines. It also raises the need for clearer thresholds in labeling content as trustworthy, especially when informing personal health decisions. Addressing these gaps could include developing a weighted scoring system that prioritizes high-risk topics, incorporates sentiment analysis to flag emotionally manipulative but inaccurate content, and uses machine learning to detect omissions. Such a tool could help platforms promote more accurate content and help viewers identify trustworthy sources.

## Conclusions

This study provides a critical evaluation of the most-viewed YouTube videos on water birth, revealing a significant disconnect between popular content and established clinical guidelines. Only a small fraction of videos accurately reflected ACOG recommendations, while the majority omitted essential safety information, often framing water birth in a "neutral" manner that masked the absence of risk discussion. This neutrality paradox, combined with the high emotional polarization of North American content, suggests that viewers may be making decisions based on incomplete or misleading narratives. While this study cannot establish causal relationships between video exposure and viewer decisions, the patterns observed warrant attention from clinicians, educators, and platform policymakers. The findings underscore an urgent need for clinicians and health organizations to collaborate with content creators to disseminate accurate, balanced, and engaging information on digital platforms. Future research should expand to multilingual content and develop more nuanced tools for evaluating risk communication and emotional framing to improve digital health literacy and support informed perinatal decision-making.

## Appendices

Appendix 1 

**Table 5:** Table of Characteristics Obtained

Metric	Details
Likes	
Views	
Amount of Comments	
Length/Duration	
Year Published	
Subscribers (at time of data collection)	
Age Restricted	Yes / No
Accredited Resource Tag	Yes / No
Thumbnail Emotion (validated chart)	(Specify emotion)
Facial Expression	Live Action / Emojis / None / Unable to Decipher
Calming	Yes / No
Tender	Yes / No
Supported/Loved/Loving	Yes / No
Emotional	Yes / No
Painful	Yes / No
Shocked/Dramatic	Yes / No
Joy	Yes / No
Neutral	Yes / No
Expressions Not Shown/Unable to Decipher	Yes / No
Text Present in Video	Yes / No + Description
Emoji/Pictograph Use	Yes / No + Examples
Affiliate Link Present	Yes / No
Doula/Midwife/Birthing Center Info	Yes / No
Link to ACOG/Reputable Resource	Yes / No
Disclosure Statement Present	Yes / No
Region Created	North America / South America / Europe / Asia / Africa / Australia / Antarctica
Video Type	Live/Vlog / Personal Recounting / Simulation / Lecture
Channel Account Association	Personal / Business (Product) / Business (Service) / Health System
Featured Content Creator	Health Professional / Midwife / Doula / Personal (Pregnant individual/family) / Company

Appendix 2 

**Table 6:** Water Birth Evidence-Based Rubric

#	Category	Criterion	Scoring	ACOG Response / Guideline Summary
1	Neonatal Diving Reflex	Discussed?	Yes / No (0)	Although it has been claimed that neonates delivered into the water do not breathe, gasp, or swallow water because of the protective “diving reflex,” experimental studies on animals and a vast body of literature from meconium aspiration syndrome demonstrate that, in compromised fetuses and neonates, the diving reflex is overridden, which potentially leads to gasping and aspiration of the surrounding fluid. Moreover, the presence of the diving reflex at birth and timing of its activation in healthy newborns have been questioned.
		If Yes:	Explanation supporting benefits = -1	
			Explanation supporting risks = +1	
			Explanation neutral (both risks and benefits) = +1	
2	Neonatal Overall Benefit	Discussed?	Yes / No (0)	No neonatal benefits associated with immersion known at this time.
		If Yes:	Explanation supporting benefits = -1	
			Explanation supporting risks = +1	
			Explanation neutral = 0	
3	Neonatal Infection Risk	Discussed?	Yes / No (0)	Risk of infection (e.g., Pseudomonas aeruginosa, Legionella pneumophila).
		If Yes:	Claims decreased/no risk of infection = -1	
			Acknowledges increased risk of infection = +1	
4	Neonatal Water Aspiration Risk	Discussed?	Yes / No (0)	Water aspiration (accompanied by hyponatremia and seizures).
		If Yes:	Claims decreased/no risk of aspiration = -1	
			Acknowledges increased risk of aspiration = +1	
5	Umbilical Cord Avulsion Risk	Discussed?	Yes / No (0)	Umbilical cord avulsion (not associated with additional neonatal morbidity, though some affected newborns required ICU admission and transfusion).
		If Yes:	Claims decreased/no risk of umbilical cord avulsion = -1	
			Acknowledges increased risk of umbilical cord avulsion = +1	
6	Maternal Overall Health Benefits	Discussed?	Yes / No (0)	No overall maternal health benefits.
		If Yes:	Explanation supporting benefits = -1	
			Explanation supporting no benefits = +1	
7	Maternal Overall Health Risks	Discussed?	Yes / No (0)	No increased maternal health risks currently known including infections and postpartum hemorrhage.
		If Yes:	Explanation claiming risks = -1	
			Explanation explaining no known health risks = +1	
8	Neonatal Risk (First Stage Labor)	Discussed?	Yes / No (0)	No increased neonatal risks that can be tied to immersion in first stage of labor.
		If Yes:	Explanation claiming risks = -1	
			Explanation explaining no known health risks = +1	
9	Method of Delivery (C-section/Assisted)	Discussed?	Yes / No (0)	No differences in need for either assisted vaginal delivery or cesarean delivery in first phase immersion.
		If Yes:	Claims higher C-sections/assisted delivery in water birth = -1	
			Claims higher C-sections/assisted delivery in traditional birth = -1	
			States no difference = +1	
10	Maternal Benefit: Less Episiotomies	Discussed?	Yes / No (0)	Maternal benefit of less episiotomies.
		If Yes:	Explaining benefit = +1	
			Claiming no benefit = -1	
11	Duration of Labor	Discussed?	Yes / No (0)	Shorter labor is seen during phase 1 for immersion, but no difference for phase 2.
		If Yes:	Claims shorter duration in phase 1/immersion = +1	
			Claims shorter duration without specifying only in phase 1 = -1	
			Claims longer duration of labor overall = -1	
12	Analgesia Use	Discussed?	Yes / No (0)	Less spinal and epidural analgesia during phase 1 for immersion.
		If Yes:	Supporting benefit by acknowledging less analgesia during phase 1 = +1	
			Supports benefit without specifying only in phase 1 = -1	
			Denies benefits = -1	
13	Maternal Satisfaction	Discussed?	Yes / No (0)	Feelings of relaxation, warmth, privacy. Improved ability to maintain control during labor.
		If Yes:	Supporting maternal satisfaction = +1	
			Denying maternal satisfaction = -1	
14	Candidate Selection	Discussed?	Yes / No (0)	Facilities that plan to offer immersion during labor and delivery need to establish rigorous protocols for candidate selection (Uncomplicated healthy 37.0-41.6 WGA).
		If Yes:	Supports having a protocol = +1	
			States protocol is not necessary = -1	
			States protocol, but protocol is incorrect = -1	
15	Infection Prevention Protocols	Discussed?	Yes / No (0)	Support having infection control procedures, including standard precautions and personal protective equipment for healthcare personnel (maintaining and cleaning of tubs and pools, infection control, PPE).
		If Yes:	Supporting having a protocol = +1	
			States no protocol necessary = -1	
16	Monitoring & Contingency Plans	Discussed?	Yes / No (0)	Monitoring of women and fetuses at appropriate intervals while immersed; and moving women from tubs if urgent maternal or fetal concerns or complications develop.
		If Yes:	Supporting monitoring for contingency plan = +1	
			Not supporting monitoring for contingency plan = -1	
